# The Deformation of a Liquid Metal Droplet Under Continuous Acceleration in a Variable Cross-Section Groove

**DOI:** 10.3390/mi15121472

**Published:** 2024-12-04

**Authors:** Hanyang Xu, Haojie Dang, Wenchao Tian, Zhao Li

**Affiliations:** School of Electro-Mechanical Engineering, Xidian University, Xi’an 710071, China; 23041110450@stu.xidian.edu.cn (H.D.); wctian@xidian.edu.cn (W.T.); zhaoli628@stu.xidian.edu.cn (Z.L.)

**Keywords:** liquid droplet MEMS switch, continuous acceleration, droplet deformation

## Abstract

This paper constructs a numerical simulation model for the deformation of droplets in a variable cross-section groove of a liquid droplet MEMS switch under different directions, amplitudes, frequencies, and waveforms of acceleration. The numerical simulation utilizes the level set method to monitor the deformation surface boundary of the metal droplets. The simulation outcomes manifest that when the negative impact acceleration on the *X*-axis is 12.9 m/s^2^, the negative impact acceleration on the *Y*-axis is 90 m/s^2^, the negative impact acceleration on the *Z*-axis is 34.5 m/s^2^, and the metal droplet interfaces with the metal electrode. The droplet deformation under the effect of a sine wave acceleration signal in the X and Y directions is lower than that under impact acceleration, while in the Z direction, the deformation is higher than that under impact acceleration. The deformation of metal droplets under square wave acceleration is more pronounced than that under sinusoidal wave acceleration. The deformation escalates with the augmentation in square wave amplitude and dwindles with the reduction in square wave acceleration frequency. Furthermore, there exists a phase difference between the deformation curve of the metal droplet and the continuous acceleration signal curve, and the phase difference is dependent of the material properties of the metal droplet. This work elucidates the deformation of the liquid-metal droplets under continuous acceleration and furnishes the foundation for the continuous operation design of MEMS droplet switches.

## 1. Introduction

Microelectromechanical system (MEMS) switches have been extensively employed in fields such as communications and consumer electronics due to their high performance, low cost, and low power consumption [1,2]. These switches are generally composed of cantilever beams and metal electrodes and will experience electron migration when the external input voltage (or current) is excessive [3]. The electron migration phenomenon in the cantilever beam and electrodes of MEMS switches increases the surface roughness of the contact area, leading to poor contact in MEMS switches, which affects the stability and service life of the switches [4]. Kondoh reported a liquid-metal droplet switch in 2005, and the switch has demonstrated long life (over 10^8^ cycles) [5]. After that, liquid-metal droplets have been extensively employed in various MEMS switch studies, such as the Galinstan droplet inertial switch reported by Liu in 2022 [6], the liquid-metal droplet RF MEMS switch reported by Rong in 2020 [7], and the gallium indium alloy droplet RF MEMS switch reported by Koo in 2015 [8]. This droplet MEMS switch realizes the “on” or “off” state of the switch through the movement or deformation of the metal droplet in a variable cross-section groove and mitigates the problems of wear, signal bouncing, and overall performance loss caused by solid–solid contact in solid-state contact switches [9,10]. Generally, the droplet MEMS switch conduction is induced by the deformation or reciprocating motion of the droplet in the groove [11]. As the speed of signal transmission heightens, the movement speed of the droplet in the groove also intensifies [12]. Consequently, comprehending the deformation of liquid-metal droplets in a variable cross-section groove under continuous driving signals will facilitate designers analyzing the performance of droplet MEMS switches.

The deformation of metal droplets within the switch groove has a direct bearing on the switch performance [13]. Through delving into the motion and deformation characteristics of liquid droplets in groove, the structure of droplet MEMS switches can be efficaciously designed and optimized. Kim first equated the motion model of metal droplets in the groove to the model of droplets breaking through capillary microvalves and furnished the working principle of capillary microvalves [14]. The research findings suggest that for finite-length rectangular capillaries, the dynamic contact angle reaches a critical value, and the contact line commences to move forward. When the droplet surface expands and approximates the critical advancing contact angle, the droplet is capable of breaking through the capillary microvalve. Based on this capillary microvalve model, Park reported on a MEMS metal droplet-based inertial micro switch in 2011. The inertial switch selectively modifies the channel surface to form microstructures, enhancing the non-wetting behavior of droplets on the surface and effectively reducing the contact angle of metal droplets within the microstructured channel [15]. The movement of metal droplets within the capillary microvalve involves droplet profile deformation. To better describe the motion process of the metal droplets, it is necessary to track the deformation profile of the droplets. The deformation of the metal droplet in capillary microvalve comprises two parts: the deformation of the metal droplet itself and the deformation of the domain around the metal droplet. Therefore, a multi-stage method is requisite to trace the connection surface of each domain in the process of numerical analysis [16,17]. Liu utilized the volume of fluid (VOF) approach to investigate the deformation of metal droplets in grooves with different hydrophobic layer thicknesses. The model of the metal droplet breaking through the capillary microvalve under inertial force is presented, and the contour of the droplet deformation process under different hydrophobic conditions in the groove is analyzed [18]. Aboutalebi resorted to numerical simulation to delve into the effects of magnetic field intensity and droplet size on the ferromagnetic droplet splitting process in symmetric T-junction [19]. Compared with the VOF multiphase method, the level set multiphase method has the ability to streamline the process of capturing topological changes caused by surface tension [20,21]. In 2021, Xu formulated the deformation model of metal droplets under acceleration in the annular groove and adopted the level set method to monitor the deformation of the surface profile of metal droplets [22]. The aforementioned study described the deformation model of metal droplets in a capillary microvalve under the action of an impact force and tracked the deformation profile of the metal droplets. However, the metal droplet MEMS switch requires the droplet to move back and forth within the groove (breaking through the capillary valve indicates the switch is open while returning to the capillary valve indicates the switch is closed). Hence, the acceleration signal applied to the metal droplet is continuous and periodically varying. Presently, the research of the metal droplet deformation under continuous acceleration in a variable cross-section groove is scarce. The relationship between the frequency, amplitude, and waveform of the continuous acceleration signal and the deformation of the metal droplet is worthy of exploration.

This paper undertakes numerical simulation research on the deformation of metal droplets in a variable cross-section groove under continuous acceleration, establishing simulation models for metal droplets in a variable cross-section groove under the influence of impact signals, sine wave signals, and square wave signals. The distribution of the metal droplet in the deformation process was explored by recourse to the laminar level set method, and the effects of different acceleration directions, amplitudes, frequencies and waveforms on the deformation of droplets were analyzed. The numerical simulation outcomes reveal that the curve of metal droplet deformation with time step bears resemblance to the continuous acceleration signal, and the deformation value of metal droplet increases with the acceleration amplitude. Nonetheless, with the augmentation of the input sine wave acceleration frequency, the deformation value of the metal droplet declines under the negative acceleration of the *X*-axis and *Y*-axis and ascends under the negative acceleration of the *Z*-axis. Additionally, the deformation curve of the metal droplet and the continuous acceleration signal curve possess a phase difference, and the phase difference is independent of the frequency of the input acceleration signal and dependent of the material properties of metal droplet. Hence, in the design of continuous driving acceleration signal, it is essential to prevent the metal droplet from cracking caused by the superposition of driving signals.

## 2. Computational Models and Methods

### 2.1. 3D Geometric Models

**Figure 1:**
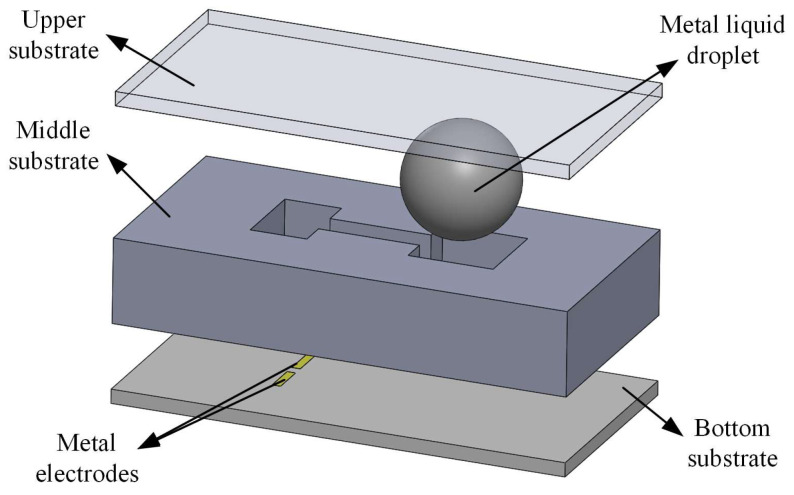
Schematic diagram of MEMS switch model.

Figure 1 demonstrates the schematic diagram of the 3D model of the MEMS switch. The model comprises an upper substrate, a middle substrate, a bottom substrate, and a liquid-metal droplet. The middle substrate features a variable cross-section groove that provides a deformation channel for the droplet, with geometric dimensions measuring 4 mm × 8 mm × 1.5 mm. This variable cross-section groove is composed of two wide reservoirs connected by a narrow groove in the middle. The thicknesses of both the bottom and upper substrates are 0.25 mm, with two metal electrodes deposited at the center of the bottom substrate’s surface. Furthermore, the upper substrate functions as a cover plate for the switch packaging.

### 2.2. Governing Equations

The inertial acceleration induces the deformation of the metal droplet, facilitating its movement into the left-side reservoir and establishing a connection between the metal electrodes to enable conduction in the MEMS switch. The inertial acceleration directed along the negative *X*-axis serves as the driving force for the MEMS switch, while accelerations in both *Y*-axis and *Z*-axis directions are classified as non-driving accelerations. Excessive acceleration in these non-driving directions may also lead to unintended conduction between the metallic droplet and metal electrodes. During this deformation process, the Reynolds number remains below 150, indicating that turbulence is not reached. Consequently, Navier–Stokes equations for incompressible laminar flow were employed to determine changes in flow velocity and pressure within the fluid [23,24].

(1) $$\rho \frac{\partial u}{\partial t}-\nabla \cdot \mu \left(\nabla u+{\left(\nabla u\right)}^{T}\right)+\rho \left(u\cdot \nabla \right)u+\nabla p=F_{st}+\rho a$$

(2) ∇⋅u=0

where **u** is the velocity vector, *ρ* is the density, *p* is the pressure, and *α* is the total acceleration of the flow, encompassing both inertial and gravitational accelerations, and *F_st_* is the surface tension.

Since the metal droplet is surrounded by air, the model defines a two-phase flow to account for both air and liquid droplets in simulation, necessitating the consideration and updating of the interfacial profile between the two phases. Therefore, the level set method was employed to solve the incompressible two-phase flow with a surface tension model. The smoothing function of level set is utilized to characterize the interface between the two phases, where the level set function equals 0 in the air phase, 1 in the droplet phase, and transitions from 0 to 1 at their interface. The level set function *ϕ* is defined as shown in Equation (3):

(3) $$\frac{\partial \phi }{\partial t}+u\cdot \nabla \phi =0$$

The unit normal vector **n** and the curvature *κ* of the interface between the two phases are presented as follows:

(4) $$n=\frac{\nabla \phi }{\left|\nabla \phi \right|}$$

(5) $$\kappa =\nabla \cdot \frac{\nabla \phi }{\left|\nabla \phi \right|}$$

Here, the scalar surface tension can be expressed as a function of the curvature of the interface:

(6) $$F_{st}=\sigma \kappa \delta \left(\phi \right)n$$

Given that the interface can be represented by level set function, it becomes feasible to investigate the alterations in the interface between the metal droplet and the air following the application of acceleration.

### 2.3. Computational Models and Boundary Conditions

**Figure 2:**
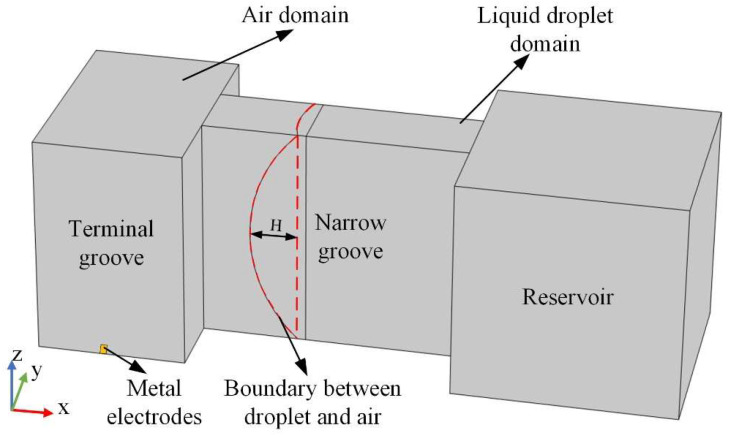
Schematic diagram of numerical model.

The numerical models of the metal droplet and the variable cross-section groove were implemented in COMSOL software 5.6. The model focused solely on the interaction between the air domain and the metal droplet domain while neglecting the structural deformation of the MEMS switch induced by acceleration. Figure 2 illustrates the initial static 3D model of the variable cross-section groove and the metal droplet. The variable cross-section groove comprised a terminal groove, a narrow groove, and a reservoir. The geometric dimensions of the groove are presented in Table 1:

Two metal electrodes were positioned at the bottom of the terminal groove and centrally located on the bottom surface. The width of the electrodes is 0.15 mm, while the separation between each electrode is 0.2 mm. The metal droplet with a diameter of 2 mm was initially positioned in the reservoir and was subsequently forced into the narrow groove by the upper and bottom substrates of the MEMS switch. As illustrated in Figure 2, the advancing meniscus of the metal droplet (represented by the red dashed line in Figure 2) serves as the boundary between the droplet and air, delineating the air domain and the liquid droplet domain. The relationship between the height (*H*) of the advancing meniscus and the contact angle *θ* between the metal droplet and the surface of the narrow groove is expressed as *H* = *R* × *sin*(*θ* − *π*/2) [6]. Additionally, the gravitational acceleration was applied in the negative *Z*-axis direction, while the inertial acceleration was applied in the negative directions of both the *X*-axis and *Y*-axis as well as the *Z*-axis.

The initial condition of the velocity vector was 0 m/s, and the pressure was set at 0 Pa within the incompressible laminar flow module. The level set function of the droplet domain was assigned a value of 1, while that for the level set function for the air domain was 0 in the level set module. Furthermore, the surface tension at the interface of two domains was taken into account, with a contact angle of 135° established between the droplet and the surface of the narrow groove.

Table 2 shows the properties of materials:

Phase transition primarily occurs within the droplet and at the interface between the droplet and air. Consequently, a specialized mesh refinement process is required for these regions. A mesh comprising 116,301 elements was generated using the free tetrahedral meshing method, achieving an average cell quality of 0.68.

## 3. Results and Discussion

### 3.1. Deformation of Metal Droplet Under the Acceleration in Driving Direction

**Figure 3:**
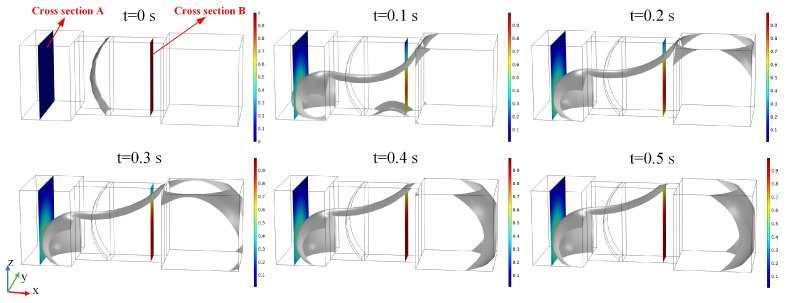
The deformation results of the droplet under negative X-direction accelerations of 12.5 m/s^2^ are presented for time steps ranging from 0 s to 0.5 s.

The negative *X*-axis direction serves as the driving direction for the droplet MEMS switch. Figure 3 illustrates the volume fractions of the liquid droplet domain and the air domain under a negative X-direction acceleration of 12.5 m/s^2^ over the time interval from 0 to 0.5 s. The gray contour represents a level set function value of 0.5, delineating the boundary of the metal droplet. Additionally, Figure 3 presents two cross sections: cross-section A is situated within the terminal groove and illustrates the profile of the metal electrode, while cross-section B is positioned within the narrow groove to depict the droplet profile. The MEMS switch was activated when the metal droplet passed through cross-section A (cross-section A = 0.58 mm).

Figure 3 indicates that in the initial state (t = 0 s), the metal droplet volume is compressed into the narrow groove, resulting in the formation of a meniscus-shaped liquid surface. When subjected to an acceleration of 12.5 m/s^2^ in the negative direction along the *X*-axis, the metal droplet migrates into the terminal groove and progressively reaches its left boundary. The advancing interface of the droplet intersects cross-section A, while cross-section B encompasses both the droplet and air domains at 0.1 s. Furthermore, after 0.4 s, the advancing interface of the liquid droplet gradually approaches a steady state.

**Figure 4:**
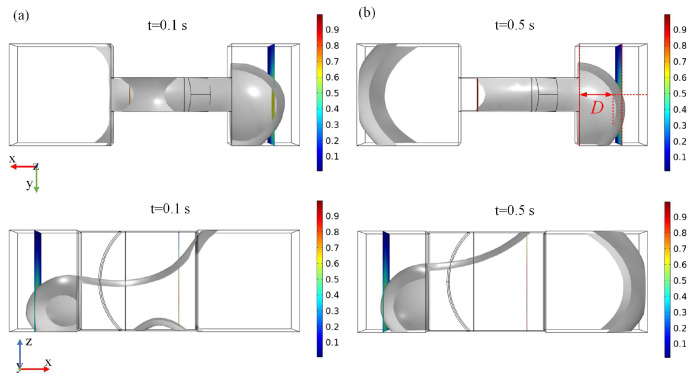
The images of droplet deformation in the XY and XZ planes under a negative *X*-axis acceleration of 12.5 m/s^2^. (**a**) is the droplet deformation at time steps of 0.1 s, (**b**) is the droplet deformation at time steps of 0.5 s.

**Figure 5:**
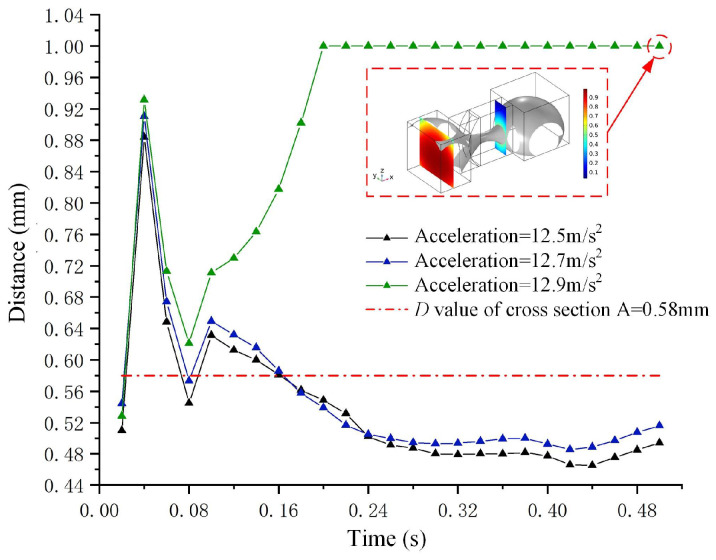
The droplet deformation curve and the input acceleration curves in the negative *X*-axis direction are presented, with the dash-dot line representing the *D* value of cross-section A.

Figure 4a,b illustrate the deformation results of the metal droplet in the XY and XZ planes, respectively, under the negative *X*-axis acceleration of 12.5 m/s^2^ at time steps of 0.1 s and 0.5 s. To facilitate the analysis of the droplet deformation, a variable termed “distance” (denoted as *D*) is introduced. *D* represents the distance between the midpoint of the liquid droplet’s advancing interface in the terminal groove and its leftmost boundary, with a value range from −1.78 mm to 1 mm. Figure 5 illustrates the values of deformation *D* for metal droplets subjected to various accelerations along the negative *X*-axis direction.

Figure 5 illustrates the variation trend of deformation *D* in response to three different accelerations along the negative *X*-axis direction. As illustrated in Figure 5, the variation trends of the deformation *D* for accelerations of 12.5 m/s^2^ and 12.7 m/s^2^ are comparable; specifically, *D* initially increases rapidly, followed by a decrease, and ultimately stabilizes at 0.48 mm. In contrast, when the acceleration reaches 12.9 m/s^2^, the value of *D* continues to increase and ultimately stabilizes at 1 mm. The red dashed box in Figure 5 highlights the deformation of the liquid droplet under an acceleration of 12.9 m/s^2^ at a time step of 0.5 s. The advancing interface of the metal droplet reaches the left boundary of the terminal groove, where the value of *D* is 1 mm. Additionally, when the value of *D* exceeds 0.58 mm, conduction occurs between the metal droplet and the metal electrodes, activating the switch.

The results presented in Figure 5 indicate that the driving acceleration required for droplet MEMS is a minimum of 12.9 m/s^2^. Furthermore, when the time step reaches 0.3 s, the value of *D* begins to stabilize, with the time step of 0.4 s considered as the steady-state time for droplet deformation.

### 3.2. Deformation of Metal Droplet Under Acceleration in Non-Driving Direction

This section presents the simulations of metal droplet deformations under acceleration in the negative *Y*-axis and negative *Z*-axis directions.

(1) Deformation results of metal droplet under Y-axis acceleration

**Figure 6:**
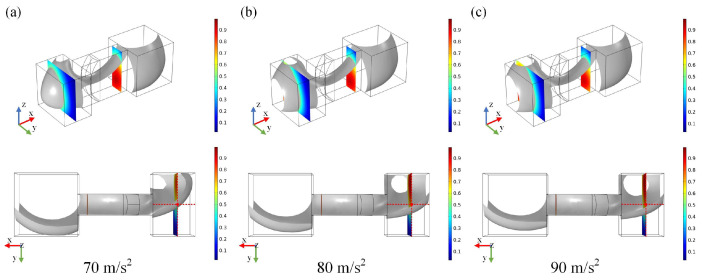
Schematic diagram of metal droplet deformation under negative *Y*-axis acceleration. (**a**–**c**) are the accelerations of 70 m/s^2^, 80 m/s^2^, and 90 m/s^2^, respectively.

Figure 6a–c illustrate the steady-state deformation (t = 0.4 s) of the metal droplet under negative *Y*-axis accelerations of 70 m/s^2^, 80 m/s^2^, and 90 m/s^2^, respectively. The advancing interface of the metal droplet traverses the narrow groove in the negative *Y*-axis direction and ultimately reaches the terminal groove under negative *Y*-axis acceleration. As the acceleration increases, the advancing interface of the metal droplet gradually approaches the metal electrode, resulting in switch conduction at an input acceleration of 90 m/s^2^. The results presented in Figure 6 indicate that the acceleration in the *Y*-axis direction has a lesser impact on the deformation of the metal droplet compared to acceleration in the *X*-axis direction.

(2) Deformation results of metal droplet under Z-axis acceleration

**Figure 7:**
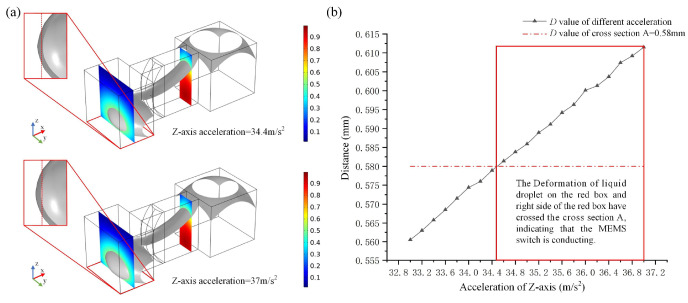
Schematic representation of metal droplet deformation under negative *Z*-axis acceleration, excluding gravitational acceleration. (**a**) is the deformation of the droplet under Z-axis negative acceleration, (**b**) is the relationship between the Z-axis accelerations and the deformation *D* value.

Figure 7a illustrates the deformation of the droplet under *Z*-axis negative acceleration of 34.4 m/s^2^ and 37 m/s^2^ (t = 0.4 s). The metal droplet is compressed into the terminal groove by the *Z*-axis negative acceleration; as the acceleration increases, the advancing interface of the metal droplet approaches section A at an acceleration of 34.4 m/s^2^ and subsequently exceeds it at an acceleration of 37 m/s^2^. Figure 7b illustrates the relationship between the *Z*-axis accelerations and the deformation *D* value, with accelerations ranging from 33 m/s^2^ to 37 m/s^2^. The deformation *D* exceeds 0.58 mm when the acceleration is ≥34.5 m/s^2^, where 0.58 mm represents the minimum *D* value required for switch conduction. The sliding of the metal droplet along the side surface of the terminal groove inhibits its movement on the bottom surface. Consequently, acceleration in the *Z*-axis direction (34.5 m/s^2^) is more likely to facilitate switch conduction compared to the acceleration in the Y-direction (90 m/s^2^).

### 3.3. Deformation of Metal Droplet Under the Action of Periodic Acceleration Signals

This section presents the simulation results of metal droplet deformation under the influence of periodic acceleration signals characterized by varying directions, amplitudes, frequencies, and waveforms.

(1) Deformation results of metal droplet subjected to sine wave acceleration in driving direction

**Figure 8:**
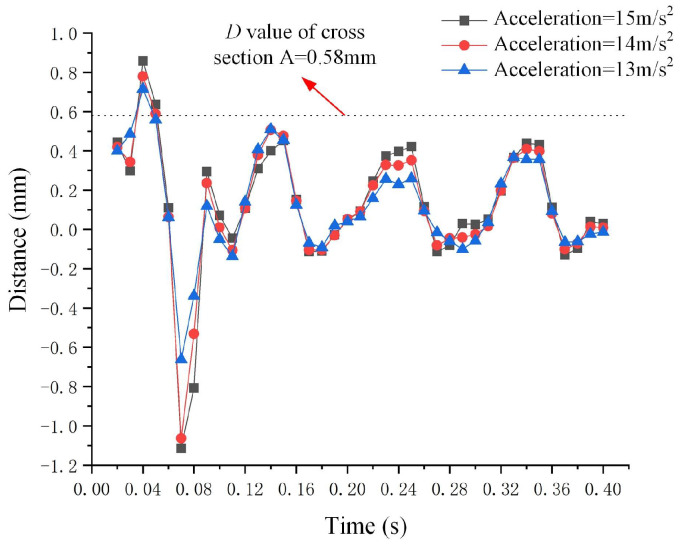
Results of metal droplet deformation under varying amplitudes of negative *X*-axis sine wave acceleration.

Figure 8 illustrates the deformation results (*D*) of the metal droplet in relation to various amplitudes of sine wave acceleration, specifically 13 m/s^2^, 14 m/s^2^, and 15 m/s^2^. The acceleration is directed along the negative *X*-axis, described by the equation *a*(*t*) = *A* × *sin*(20*π* × *t*), where *A* represents the amplitude and *t* denotes the time step. The deformation *D* of the metal droplet exhibits significant changes before 0.1 s, indicating that it is in a non-steady state; conversely, *D* tends to stabilize when the time step exceeds 0.1 s, signifying that the metal droplet has reached a steady state. However, when the amplitude of the sine wave acceleration reaches 15 m/s^2^, the deformation *D* of the metal droplet in a steady state remains below 0.58 mm.

**Figure 9:**
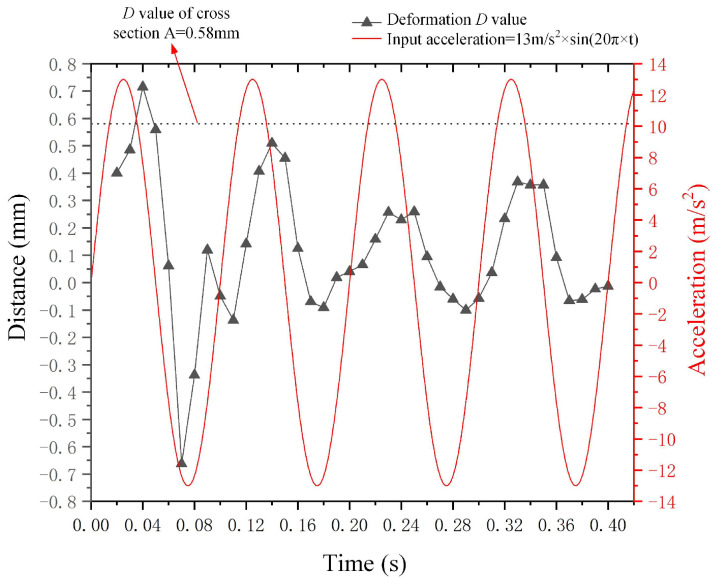
Curves depicting the droplet deformation *D* and input sine wave acceleration.

Additionally, the trend of droplet deformation *D* in relation to the input sine wave acceleration signal is presented. Figure 9 illustrates the curves of both the input negative *X*-axis sine wave acceleration signal and the corresponding deformation *D* of the metal droplet. The acceleration equation is given by the following: *a*(*t*) = 13 m/s^2^ × *sin*(20*π* × *t*), and the curve representing metal droplet deformation *D* exhibits a similar trend to that of the input signal curve in both the non-steady state (t ≤ 0.1 s) and steady state (t > 0.1 s). The viscous properties of the metal droplets induce a lag in deformation relative to the input signal, leading to a phase difference between the deformation *D*-value curve and the sine wave acceleration input curve.

(2) Results of metal droplet deformation under the influence of sine wave acceleration in non-driving direction

- Results of metal droplet deformation under sine wave acceleration along Y-axis direction

**Figure 10:**
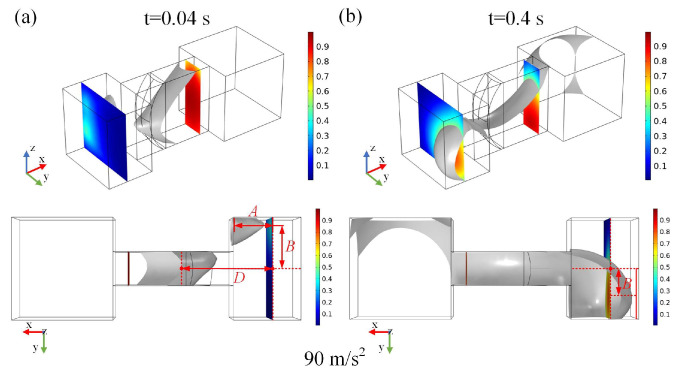
Results of metal droplet deformation under *Y*-axis sine wave acceleration. (**a**) is the deformation result at time step of 0.04 s, (**b**) is the result at time step of 0.4 s.

Figure 10a,b illustrate the deformation results of the metal droplet at different time steps under the influence of negative *Y*-axis sine wave acceleration with time steps of 0.04 s and 0.4 s, described by the equation *a*(*t*) = 90 m/s^2^ × *sin*(20*π* × *t*). At the time step of 0.04 s, the metal droplet within the narrow groove is compressed, resulting in a small volume being confined to the terminal groove where it contacts the negative XZ plane. As the time step increases to 0.4 s, a large volume of metal droplets is forced into the terminal groove, making contact with its positive XZ plane.

Additionally, variables *A* and *B* are defined to quantitatively describe the deformation results of metal droplet. Variable *A* is the distance between the midpoint of the forefront liquid surface of the metal droplet in the XY plane and the left end of the terminal groove, while variable *B* denotes the distance between the midpoint of the forefront liquid surface of the droplet in the XY plane and the horizontal midpoint of the terminal groove.

**Figure 11:**
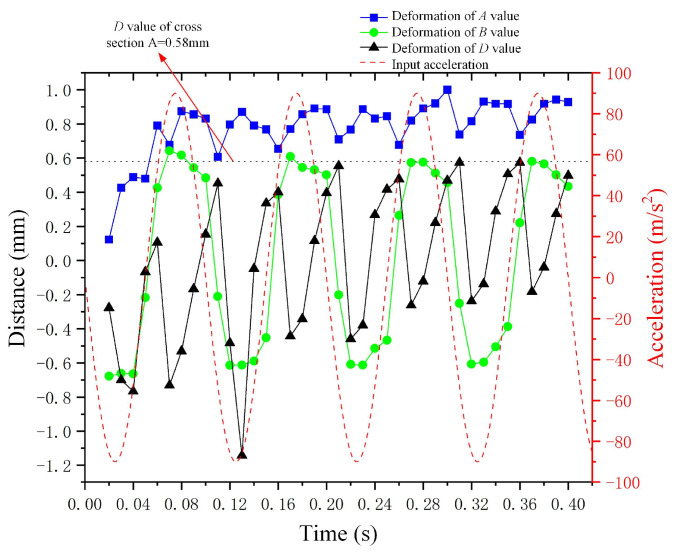
The correlation between input negative *Y*-axis sine wave acceleration and the deformation of metal droplets.

Figure 11 illustrates the curve of the sine wave acceleration (described by the equation: *a*(*t*) = 90 m/s^2^ × *sin*(20*π* × *t*)) alongside the deformation curves for variables *A*, *B*, and *D* of the metal droplet. The variation in deformation *A* is relatively minor, remaining largely stable within the range of 0.6 mm to 1 mm. The deformation *B* of the metal droplet exhibits a trend and periodicity that closely align with those of the input sine wave acceleration. However, the period of deformation *D* is significantly larger than that of the input sine wave acceleration, indicating that the forefront liquid surface of the metal droplet is more responsive than the advancing interface. Furthermore, the value of deformation *D* remains below 0.58 mm when subjected to a negative *Y*-axis sine wave acceleration represented by *a*(*t*) = 90 m/s^2^ × *sin*(20*π* × *t*).

- Results of metal droplet deformation under Z-axis sine wave acceleration

**Figure 12:**
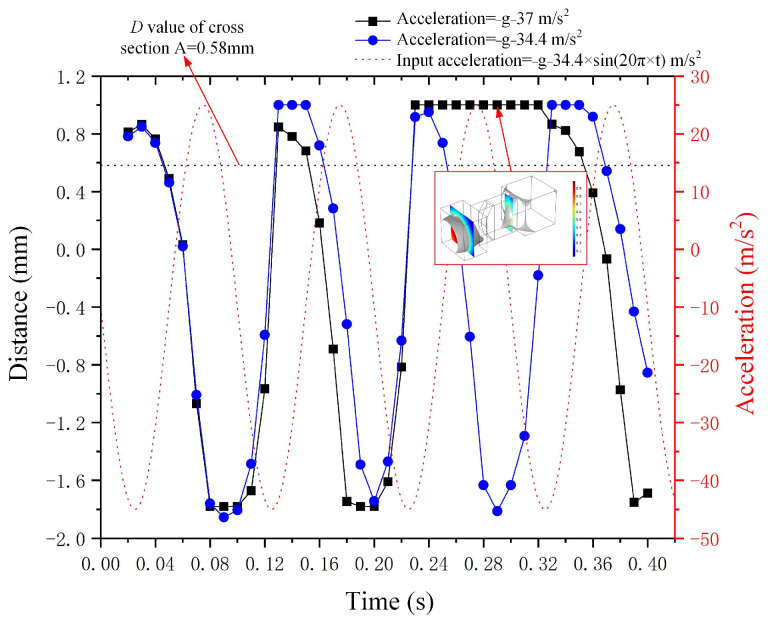
The correlation between input negative *Z*-axis sine wave acceleration and the deformation of metal droplets.

Figure 12 illustrates the curve of deformation *D* value for the metal droplet alongside the curves of sine wave acceleration with varying amplitudes in the negative direction of the *Z*-axis. The equations for these sine wave accelerations are *a*(*t*) = −*g* − 34.4 m/s^2^ × *sin*(20*π* × *t*) and *a*(*t*) = −*g* − 37 m/s^2^ × *sin*(20*π* × *t*), where *g* represents gravitational acceleration. The curve of deformation *D* exhibits a similarity to the input sine wave acceleration, characterized by a phase difference. Furthermore, the metal droplet within the narrow groove is observed to have been divided into two parts when the amplitude reaches 37 m/s^2^ and the time step is 0.29 s, as illustrated in Figure 12. The deformation value *D* for acceleration amplitudes of 34.4 m/s^2^ and 37 m/s^2^ yields distinct results between 0.24 s and 0.32 s.

### 3.4. Deformation of Metal Droplets Under Varying Frequency Sine Wave Acceleration Signals

**Figure 13:**
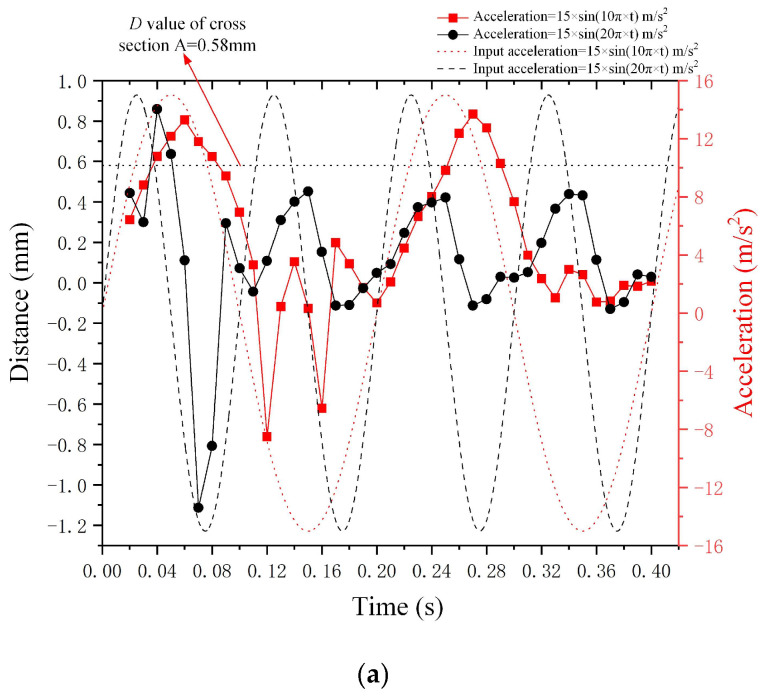

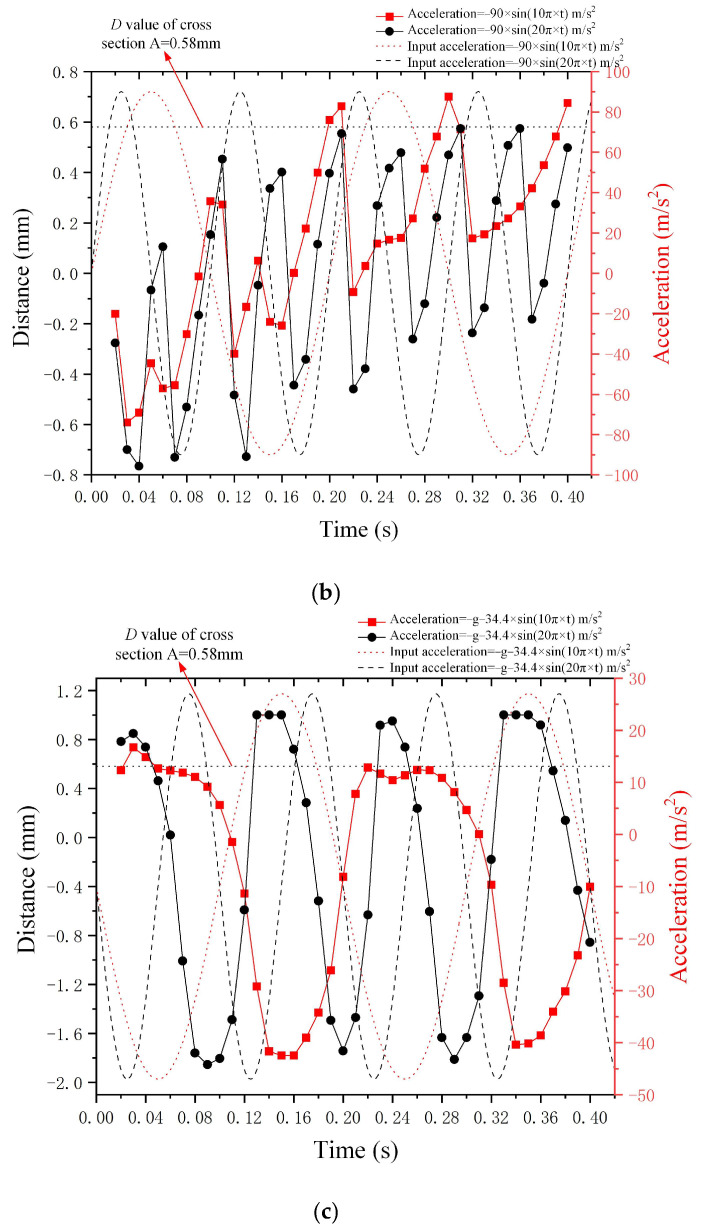
Results of sine wave acceleration curve and the metal droplet deformation curve at various frequencies. (**a**) represents acceleration in negative *X*-axis direction, (**b**) denotes acceleration in negative *Y*-axis direction, and (**c**) illustrates acceleration in negative *Z*-axis direction.

Figure 13a–c illustrate the deformation results of metal droplets subjected to sine wave signals with frequencies of 5 Hz and 10 Hz in the negative *X*-axis direction, negative *Y*-axis direction, and negative *Z*-axis direction, respectively. The red dotted line denotes the deformation value *D* of the metal droplet subjected to a sine wave acceleration with a frequency of 5 Hz, while the red dashed line represents the corresponding sine wave acceleration curve at this frequency. The black dotted line and black dashed line denote the deformation value *D* of the metal droplet subjected to a sine wave acceleration with a frequency of 10 Hz, while the latter represents the corresponding input sine wave acceleration curve at this frequency.

The deformation value *D* of the metal droplet subjected to sine wave acceleration in the negative *X*-axis and negative *Y*-axis directions exhibits a significant magnitude at low frequencies (5 Hz). Conversely, the deformation value *D* of the metal droplet in the negative *Z*-axis direction increases with frequency. At high frequencies (10 Hz), the deformation value *D* of the metal droplet subjected to acceleration along the *X*-axis and *Y*-axis does not exceed 0.58 mm; however, at a frequency of 5 Hz, this deformation value surpasses 0.58 mm. Furthermore, as illustrated in Figure 13a, the sine wave acceleration curve with a frequency of 5 Hz in the negative *X*-axis direction exhibits a phase difference relative to the deformation curve of the metal droplet, with this phase difference value being comparable to that observed at a frequency of 10 Hz. These results suggest that varying frequency sine wave signals have less impact on the phase difference between the input signal curve and the deformation *D* curve of the metal droplet.

### 3.5. Deformation of Metal Droplet Under the Square Wave Acceleration

(1) Outcomes of droplet deformation under the influence of square wave acceleration with varying amplitudes and frequencies along the *X*-axis

**Figure 14:**
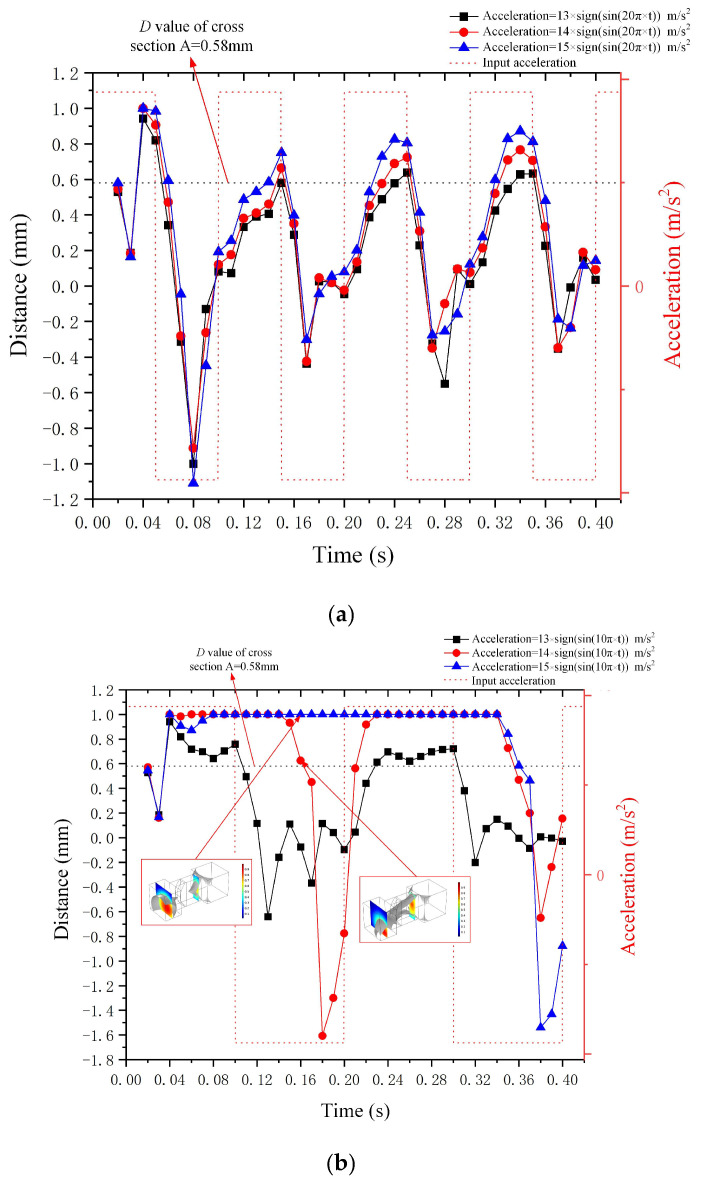
Outcomes of droplet deformation under the influence of square wave acceleration in the *X*-axis. (**a**) represents the acceleration frequency of 10 Hz, and (**b**) represents the acceleration frequency of 5Hz.

Figure 14a presents the deformation *D* value of the metal droplet subjected to the square wave accelerations with a frequency of 10 Hz in the negative *X*-axis direction. Respectively, the amplitudes of the square wave acceleration are 13 m/s^2^, 14 m/s^2^, and 15 m/s^2^. It is observable from Figure 14a that as the acceleration amplitude increases, the deformation *D* value of the metal droplet rises. Moreover, the square wave acceleration can induce a greater deformation of the metal droplet compared to the sine wave acceleration (when the acceleration amplitude reaches 13 m/s^2^, the *D* value of the steady-state deformation of the metal droplet exceeds 0.58 mm, and the switch is turned on). Furthermore, when the input square wave signal transits from “high” to “low”, the deformation *D*-value curve of the metal droplet will change simultaneously without any time lag.

Figure 14b presents the deformation *D* value of the metal droplet subjected to the square wave accelerations with varying amplitudes and a frequency of 5 Hz in the negative *X*-axis direction. The reduction in the frequency of the input acceleration prolongs the action time of the “high” and “low” accelerations, significantly enhancing the deformation *D* value of metal droplet. Additionally, as the frequency declines, the time step for the droplet to return from the terminal groove to the narrow groove raises. The red box in Figure 14b presents the results of droplet deformation with a time step of 0.16 s and amplitudes of 15 m/s^2^ and 14 m/s^2^, respectively. The metal droplet is squeezed and broken in the narrow groove when the amplitude reaches 15 m/s^2^. The metal droplets trapped in the terminal groove have received the next “high” signal input prior to moving to the narrow groove under the influence of the “low” signal input. Consequently, the deformation *D* value of the metal droplet remains unaltered under the action of the “low” signal.

(2) Outcomes of droplet deformation under the influence of square wave acceleration with varying amplitudes and frequencies in the *Z*-axis

**Figure 15:**
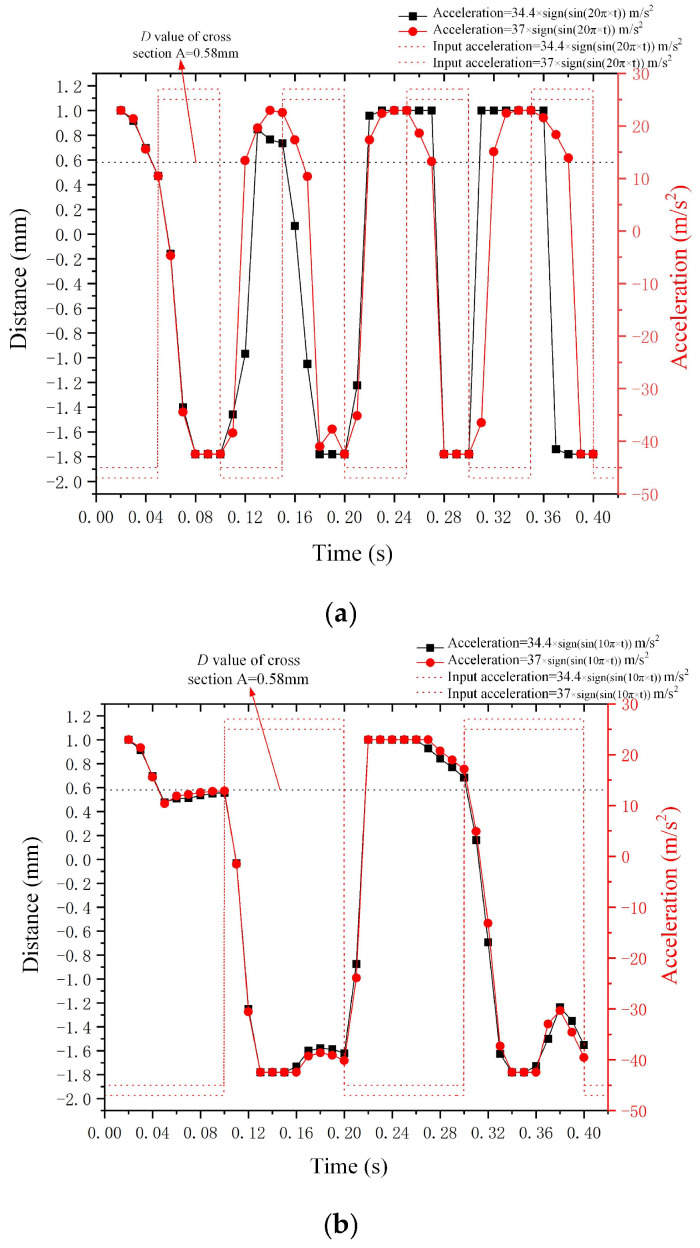
Outcomes of droplet deformation under the influence of square wave acceleration in the *Z*-axis. (**a**) represents the acceleration frequency of 10 Hz, and (**b**) represents the acceleration frequency of 5 Hz.

Figure 15a,b display the deformation outcomes of the metal droplet under *Z*-axis square wave acceleration with different amplitudes and frequencies of 10 Hz and 5 Hz, respectively (the input square wave acceleration incorporates gravitational acceleration). The deformation induced by the square wave acceleration in the *Z*-axis direction is analogous to the deformation caused by the acceleration in the *X*-axis direction. The greater the acceleration amplitude, the larger the deformation of the metal droplet. Additionally, Figure 15b indicates that the deformation *D*-value curve of the metal droplet is closer to the square wave acceleration curve in low-frequency input signal.

### 3.6. Deformation of Galinstan Droplet Under Square Wave Acceleration and Mercury Droplet Under High-Frequency Square Wave Acceleration

This section presents the outcome regarding the deformation under square wave acceleration and that of mercury droplets under high-frequency square wave acceleration.

(1) Deformation outcomes of the Galinstan droplet under square wave acceleration

Table 3 presents the material properties of the Galinstan droplet domain and the air domain [25].

**Figure 16:**
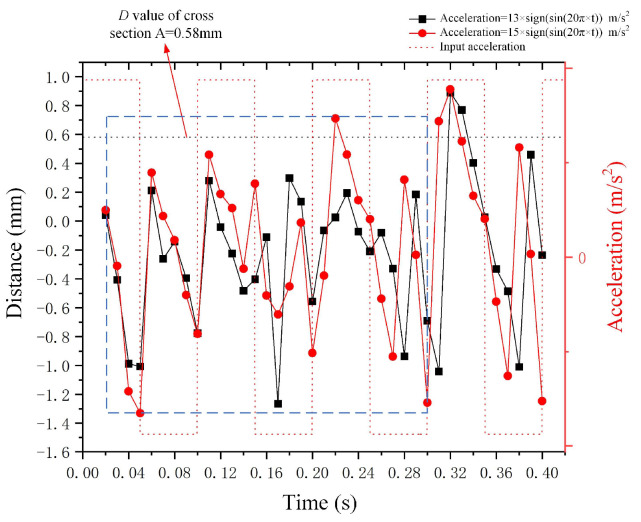
Outcomes of Galinstan droplet deformation under the impact of square wave acceleration along the *X*-axis.

Figure 16 demonstrates the deformation *D* value of the Galinstan droplet under the square wave accelerations along the negative *X*-axis. The equations of these square wave accelerations are *a*(*t*) = 13 m/s^2^ × *sin*(20*π* × *t*) and *a*(*t*) = 15 m/s^2^ × *sin*(20*π* × *t*). In comparison with mercury droplets, the viscosity and surface tension of Galinstan rise, while the density reduces. The deformation *D* value of the Galinstan droplet is unstable within the time step ranging from 0.01 s to 0.3 s (the blue dotted wireframe in Figure 16). Conversely, when the time step exceeds 0.3 s, *D* tends to stabilize, and a phase difference exists between the deformation *D* curve and the input acceleration curve, and the magnitude of the phase difference differs from that when the metal droplet is mercury. The Galinstan droplet with large surface tension and viscosity material parameters is less sensitive to deformation than mercury droplet, and the Galinstan droplet is more prone to deformation at high acceleration amplitudes. The *D* curve for the stable state at an acceleration amplitude of 13 m/s^2^ lags behind the *D* curve for the stable state at an acceleration amplitude of 15 m/s^2^. Additionally, when the Galinstan droplet is unstable, the amplitude of 13 m/s^2^ acceleration is unable to cause the value of *D* to exceed 0.58 mm. The phase difference between the deformation *D* curve and the input acceleration curve of the metal droplet of various materials varies.

(2) Deformation outcomes of mercury droplet under the high-frequency square wave acceleration

**Figure 17:**
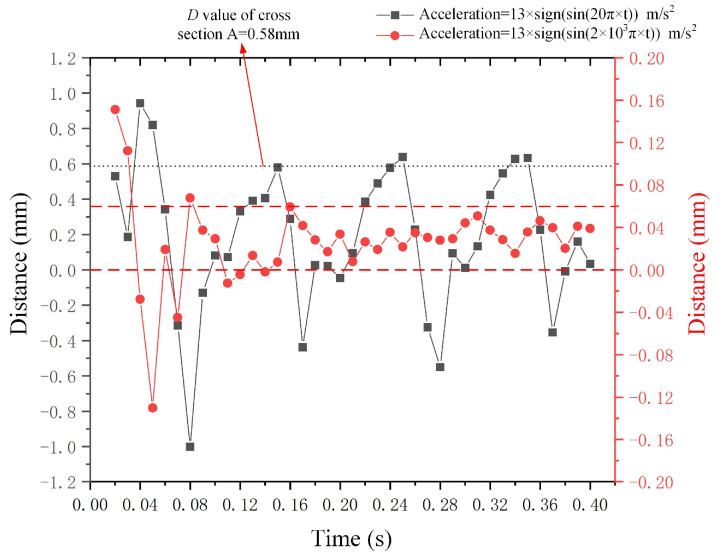
Outcomes of mercury droplet deformation under the impact of high-frequency square wave acceleration along the *X*-axis.

Figure 17 depicts the deformation *D* value of the mercury droplet under the square wave accelerations along the negative *X*-axis, and the frequencies of the acceleration signal are 10 Hz and 1000 Hz. The metal droplet attains a stable state after 0.08 s under the effect of acceleration in the negative *X*-axis direction. If the frequency of the acceleration is excessively high, the metal droplet will receive the next driving signal before completing the deformation of the current driving signal, resulting in a reduction in the deformation *D* value of the metal droplet (the deformation *D* value stabilizes between 0 mm and 0.06 mm when the frequency of the acceleration is 1000 Hz).

## 4. Conclusions

This paper proposes a model of metal droplet deformation within a variable cross-section groove subjected to a continuous acceleration signal and exhibits the simulation results of metal droplet deformation with different acceleration directions, amplitudes, frequencies, and waveforms. The following conclusions are derived:

(1) The metal droplet makes contact with the metal electrodes when the negative impact acceleration of the *X*-axis exceeds 12.9 m/s^2^, the negative impact acceleration of the *Y*-axis exceeds 90 m/s^2^, and the negative impact acceleration of the *Z*-axis exceeds 34.5 m/s^2^. The deformation of the metal droplet tends to stabilize at 0.4 s and escalates with the increase in impact acceleration. Furthermore, the variable cross-section groove, comprising the terminal groove, narrow groove, and reservoir, demonstrates an effective resistance to the acceleration in the *Y*-axis direction.

(2) The deformation curve of the metal droplet under the influence of sine wave acceleration is of a sine wave pattern. The negative *X*-axis sine wave acceleration with an amplitude of 13 m/s^2^ and the negative *Y*-axis sine wave acceleration with an amplitude of 90 m/s^2^ will not induce the conduction of metal droplets and metal electrodes (the frequency of the sine wave acceleration is 10 Hz). However, when the amplitude of the negative *Z*-axis sine wave acceleration amounts to 34.4 m/s^2^ and the frequency attains 10 Hz, the droplet interfaces with the metal electrodes. Additionally, as the frequency of the input sine wave acceleration escalates, the deformation *D* of the metal droplet diminishes under negative acceleration in the X and Y axes and augments under negative acceleration in the Z axis.

(3) The deformation curve of the metal droplet under the influence of square wave acceleration assumes the square wave pattern, and the deformation *D* value of the metal droplet ascends with the augmentation of the square wave amplitude. As the frequency of the square wave acceleration declines, the time step for the droplet to revert from the terminal groove to the narrow groove increases. Furthermore, as the frequency of the square wave increases, the metal droplet receives the next driving signal before completing the deformation of the current driving signal, and the deformation *D* value decreases.

(4) The excessive amplitude and duration of the continuous acceleration signal will result in the rupture of metal droplets in a narrow groove. The metal droplet entering the terminal groove fails to return to the narrow groove under the influence of the subsequent acceleration signal. The deformation *D* value of the metal droplet curve varies from the input acceleration signal curve when the metal droplet ruptures. Additionally, a phase difference exists between the deformation curve of the metal droplet and the continuous acceleration signal curve, and this phase difference is independent of the frequency of the input acceleration signal (when metal droplet is mercury) and is related to the material properties of the metal droplet (viscosity and surface tension) and the acceleration amplitude (when metal droplet is Galinstan). Hence, in the design of a continuous driving acceleration signal, it is essential to prevent the rupture of metal droplets caused by the superposition of driving signals.

This paper investigates the deformation of metal droplets within a variable cross-section groove under the influence of *X*-axis, *Y*-axis, and *Z*-axis continuous acceleration signals and provides a basis for the design of the continuous driving signal of the droplet MEMS switch.

## Figures and Tables

**Table 1:** Geometric dimensions of variable cross-section groove.

	Height (mm)	Length (mm)	Width (mm)
Terminal groove	1.5	1	1.5
Narrow groove	1.5	1.78	0.5
Reservoir	1.5	1.5	1.5

**Table 2:** The properties of materials.

	Materials	
Properties	Mercury (Droplet Domain)	Air (Air Domain)
Density (kg/m^3^)	13600	1.3
Viscosity (Pa.s)	1.526 × 10^−3^	1.79 × 10^−5^
Surface tension (N/m)	0.48	

**Table 3:** The properties of Galinstan and air.

	Materials	
Properties	Galinstan (Droplet Domain)	Air (Air Domain)
Density (kg/m^3^)	6440	1.3
Viscosity (Pa·s)	2.4 × 10^−3^	1.79 × 10^−5^
Surface tension (N/m)	0.605	

## Data Availability

Data are contained within the article.

## References

[1] Belkadi N., Nadaud K., Hallepee C. (2020). Zero-level packaged RF-MEMS switched capacitors on glass substrates. J. Microelectromech. Syst..

[2] Chand C.G., Maity R., Maity N.P. (2021). Electromagnetic modelling and analysis of RF MEMS capacitive shunt switch for 5G applications. Microelectron. J..

[3] Kumar M., Mukherjee B., Sen S. (2021). Analysis of static charge induced pull-in of an electrostatic MEMS. Commun. Nonlinear Sci. Numer. Simul..

[4] Liu Y., Liu J., Yu B., Liu X. (2018). A compact single-cantilever multicontact RF-MEMS switch with enhanced reliability. IEEE Microw. Wirel. Compon. Lett..

[5] Kondoh Y., Takenaka T., Hidaka T. (2005). High-reliability, high-performance RF micromachined switch using liquid metal. J. Microelectromech. Syst..

[6] Liu Z.H., Wu M.X., Xu Z., Xiang X.Y., Li H.R., Du L.Q., Liu J.S. (2022). A reusable pseudo-liquid-to-solid inertial switch based on hetero-coated Galinstan droplets. J. Microelectromech. Syst..

[7] Zhu X., Yang F., Zhao S., Wang H., Niu C., Rong M. (2020). Liquid-metal capillary switch for electrical power application. Appl. Phys. Lett..

[8] Koo C., Leblanc B.E., Kelley M., Fitzgerald H., Huff G., Han A. (2015). Manipulating liquid metal droplets in microfluidic channels with minimized skin residues toward tunable RF applications. J. Microelectromech. Syst..

[9] Bono S., Nakai R., Konishi S. (2024). Simultaneous detection of the shuttling motion of liquid metal droplets in channels under alternating pressure and capacitive sensor signals. Microsyst. Nanoeng..

[10] Xu J.S., Wang X.C., Huang Q.Y., He X.D. (2023). Droplet manipulation on an adjustable closed-open digital microfluidic system utilizing asymmetric EWOD. Lab A Chip.

[11] Sen P., Kim C.J. (2009). A fast liquid-metal droplet microswitch using EWOD-driven contact-line sliding. J. Microelectromech. Syst..

[12] Shayunusov D., Eskin D., Zeng H., Nikrityuk P.A. (2024). Behavior of small water droplets in a highly viscous flow in a converging and diverging channel. Phys. Fluids.

[13] Zhao Y., Zhang B., Lv C. (2024). Manipulation of liquid transport and droplet switch using light-actuated surface tension. Colloids Surf. A Physicochem. Eng. Asp..

[14] Cho H., Kim H.Y., Kang J.Y., Kim T.S. (2007). How the capillary burst microvalve works. J. Colloid Interface Sci..

[15] Yoo K., Park U., Kim J. (2011). Development and characterization of a novel configurable MEMS inertial switch using a microscale liquid-metal droplet in a microstructured channel. Sens. Actuators A-Phys..

[16] Chowdhary S., Reddy S.R., Banerjee R. (2020). Detailed numerical simulations of unequal sized off-centre binary droplet collisions. Int. J. Multiph. Flow.

[17] Yang H., Xu Y., Knowles T. (2023). Droplet dynamics in asymmetric microfluidic junctions. Eur. J. Mech. B-Fluids.

[18] Liu T., Su W., Yang T., Xu Y. (2014). Vibration interference analysis and verification of micro-fluidic inertial switch. AIP Adv..

[19] Aboutalebi M., Bijarchi M.A., Shafii M.B., Hannani S.K. (2018). Numerical investigation on splitting of ferrofluid microdroplets in T-junctions using an asymmetric magnetic field with proposed correlation. J. Magn. Magn. Mater..

[20] Patil N.D., Gada V.H., Sharma A., Bhardwaj R. (2016). On dual-grid level-set method for contact line modeling during impact of a droplet on hydrophobic and superhydrophobic surfaces. Int. J. Multiph. Flow.

[21] Shao C.X., Yuan S., Luo K. (2023). A generalized coupled level set/volume-of-fluid/ghost fluid method for detailed simulation of gas-liquid flows. J. Comput. Phys..

[22] Xu H.Y., Zhao Y., Zhang K. (2020). A deformation of a mercury droplet under acceleration in an annular groove. Biosensors.

[23] Pasandideh-Fard M., Aziz S.D., Chandra S., Mostaghimi J. (2001). Cooling effectiveness of a water drop impinging on a hot surface. Int. J. Heat Fluid Flow.

[24] Deshpande K.B., Smith D., Zimmerman W.B.J. (2006). Modeling of multi-phase flow using the level set method. Multiphys. Model. Finite Elem. Methods.

[25] Handschuh-Wang S., Stadler F.J., Zhou X.C. (2021). Critical review on the physical properties of Gallium-based liquid metals and selected pathways for their alteration. J. Phys. Chem. C.

